# Promoting children's sleep health: Intervention Mapping meets Health in All Policies

**DOI:** 10.3389/fpubh.2022.882384

**Published:** 2022-11-16

**Authors:** Laura S. Belmon, Maartje M. Van Stralen, Irene A. Harmsen, Karen E. Den Hertog, Robert A. C. Ruiter, Mai J. M. Chinapaw, Vincent Busch

**Affiliations:** ^1^Department of Public and Occupational Health, Amsterdam University Medical Centers (UMC), Vrije Universiteit Amsterdam, Amsterdam Public Health Research Institute, Amsterdam, Netherlands; ^2^Sarphati Amsterdam, Public Health Service (GGD), Amsterdam, Netherlands; ^3^Department of Healthy Living, Public Health Service (GGD), Amsterdam, Netherlands; ^4^Department of Health Sciences, Faculty of Science and Amsterdam Public Health Research Institute, Vrije Universiteit Amsterdam, Amsterdam, Netherlands; ^5^Department of Work and Social Psychology, Maastricht University, Maastricht, Netherlands

**Keywords:** sleep, Intervention Mapping, program development, Health in All Policies (HiAP), policy, children, childhood, intervention development

## Abstract

**Background:**

To design a comprehensive approach to promote children's sleep health in Amsterdam, the Netherlands, we combined Intervention Mapping (IM) with the Health in All Policies (HiAP) perspective. We aimed to create an approach that fits local infrastructures and policy domains across sectors.

**Methods:**

First, a needs assessment was conducted, including a systematic review, two concept mapping studies, and one cross-sectional sleep diary study (IM step 1). Subsequently, semi-structured interviews with stakeholders from policy, practice and science provided information on potential assets from all relevant social policy sectors to take into account in the program design (HiAP and IM step 1). Next, program outcomes and objectives were specified (IM step 2), with specific objectives for policy stakeholders (HiAP). This was followed by the program design (IM step 3), where potential program actions were adapted to local policy sectors and stakeholders (HiAP). Lastly, program production (IM step 4) focused on creating a multi-sector program (HiAP). An advisory panel guided the research team by providing tailored advice during all steps throughout the project.

**Results:**

A blueprint was created for program development to promote children's sleep health, including a logic model of the problem, a logic model of change, an overview of the existing organizational structure of local policy and practice assets, and an overview of policy sectors, and related objectives and opportunities for promoting children's sleep health across these policy sectors. Furthermore, the program production resulted in a policy brief for the local government.

**Conclusions:**

Combining IM and HiAP proved valuable for designing a blueprint for the development of an integrated multi-sector program to promote children's sleep health. Health promotion professionals focusing on other (health) behaviors can use the blueprint to develop health promotion programs that fit the local public service infrastructures, culture, and incorporate relevant policy sectors outside the public health domain.

## Introduction

Healthy sleep is vital for children's psychosocial and physical health (1), and preventing non-communicable diseases such as diabetes (2) and cardiovascular disease in later in life (3). Sleep health is a five-domain concept, consisting of, sleep duration [i.e., 9 h of sleep or more for children aged 6–13 years old (4)], regular sleep timing, good sleep efficiency (i.e., ease of falling and staying asleep), sleep quality, and daytime wakefulness (5). Currently, many children experience poor sleep health, with declining sleep duration in the past decades (6) and poor sleep efficiency and sleep quality being widely prevalent (7–9). Promoting children's healthy sleep is therefore an important public health challenge (10).

Thus far, programs that aimed to promote children's healthy sleep have shown only minor effects, if any (11). A reason for this may be the lack of a systematic, evidence-based program development process (11, 12), which is crucial for a program to target relevant determinants *via* appropriate behavior- or environmental change techniques. Another reason may be that previous programs were insufficiently adapted to fit targeted policy infrastructures. Sustainable implementation requires in-depth knowledge of the existing local context. Without an in-depth development process, the program may neglect the complexity of the health problem and interrelated factors, which reduces the chances of sustainable success (12). An additional reason for programs' limited success thus far may be the lack of community stakeholder participation during program development and implementation, which is key in achieving program acceptability and appropriateness (12, 13). Lastly, the lack of rigid epidemiological evaluations has thus far made it impossible to distill (in)effective elements from existing programs (11, 12, 14).

Intervention Mapping (IM) provides a framework for systematically developing health promotion programs (12). IM is specifically designed to achieve sustainable success within complex systems. It also provides support to program developers to create programs that are grounded in theory and evidence. It further encourages taking into account the system related to the health problem and considers potential interrelationships between factors, activities, stakeholders, and settings. Overall, IM provides guidance to include the most important determinants of the health problem in the relevant social-ecological context and to target these determinants *via* appropriate methods, as one coordinated whole. A noteworthy strength of this approach is that it emphasizes the importance of community stakeholder participation throughout the research, development, and implementation process of the program. Although IM will create a rich overview of the complexity of the health problem and promotes community participation in the creation of intervention actions, it does not necessarily promote sustainable impact.

To create sustainable and positive impact on children's sleep health within the complex system children live in, higher socio ecological levels (e.g., policy/society) need to be taken into account. One way to do this is by taking a Health in All Policies (HiAP) perspective (15), which may bring additional determinants, stakeholders, and assets across public policies to light. HiAP promotes active and systematic incorporation of health across important policy sectors (16). Public policy is the foundation for most health promotion work (17) and some previous health promoting programs that include policy action(s), to positively stimulate the underlying determinants, have shown to be effective (18). Furthermore, many of the determinants that influence health are not solely influenced by the public health and health care policy sectors. Therefore, instead of a program implemented in or by solely one policy sector, coordinated efforts on all relevant public policies (e.g., education, social security, and living environment) are needed to create impact on the system as one integrated multi-sector approach to optimally promote children's sleep health.

This paper describes a blueprint for the development of an integrated multi-sector program to promote children's sleep health in Amsterdam, the Netherlands, using a novel approach combining IM with HiAP.

## Materials and methods

### The Amsterdam Healthy Sleep Project

The Amsterdam Healthy Sleep Project started in 2016 as a collaboration between the Amsterdam University Medical Centers (Amsterdam UMC, location VU University Medical Center), the Public Health Service of Amsterdam, and the Vrije Universiteit Amsterdam. One researcher (LB) was appointed at two organizations (Amsterdam UMC and Public Health Service of Amsterdam) and closely involved with two other organizations. One organization was “The Amsterdam Healthy Weight Approach” of the City of Amsterdam, which is a long-term municipal health-promoting program that reaches into every domain of a child's life (19). The second organization was “Sarphati Amsterdam” research institute, which is a collaboration between the City of Amsterdam and the five knowledge institutes in Amsterdam. These organizations are part of the public health sector, and initiated this project since no programs to promote children's sleep existed in Amsterdam yet. The managers of the two latter organizations were closely involved, regularly updated by the research team, and their feedback was taken into account throughout the project. The overall project aim was to systematically develop a program for promoting healthy sleep among primary school children in Amsterdam using IM. The public health sector chose a focus on primary school aged children (4–12 years in the Netherlands) since many policy- and practice organizational structures, as well as local city council orders (i.e., local governmental decisions and agreements), are organized around the schools systems and the local political priority at the time was focused on primary school children. All studies that were part of this project were approved by the Medical Ethics Committee of the VU University Medical Center (protocol no. 2017.013 and 2018.170).

**Figure 1:**
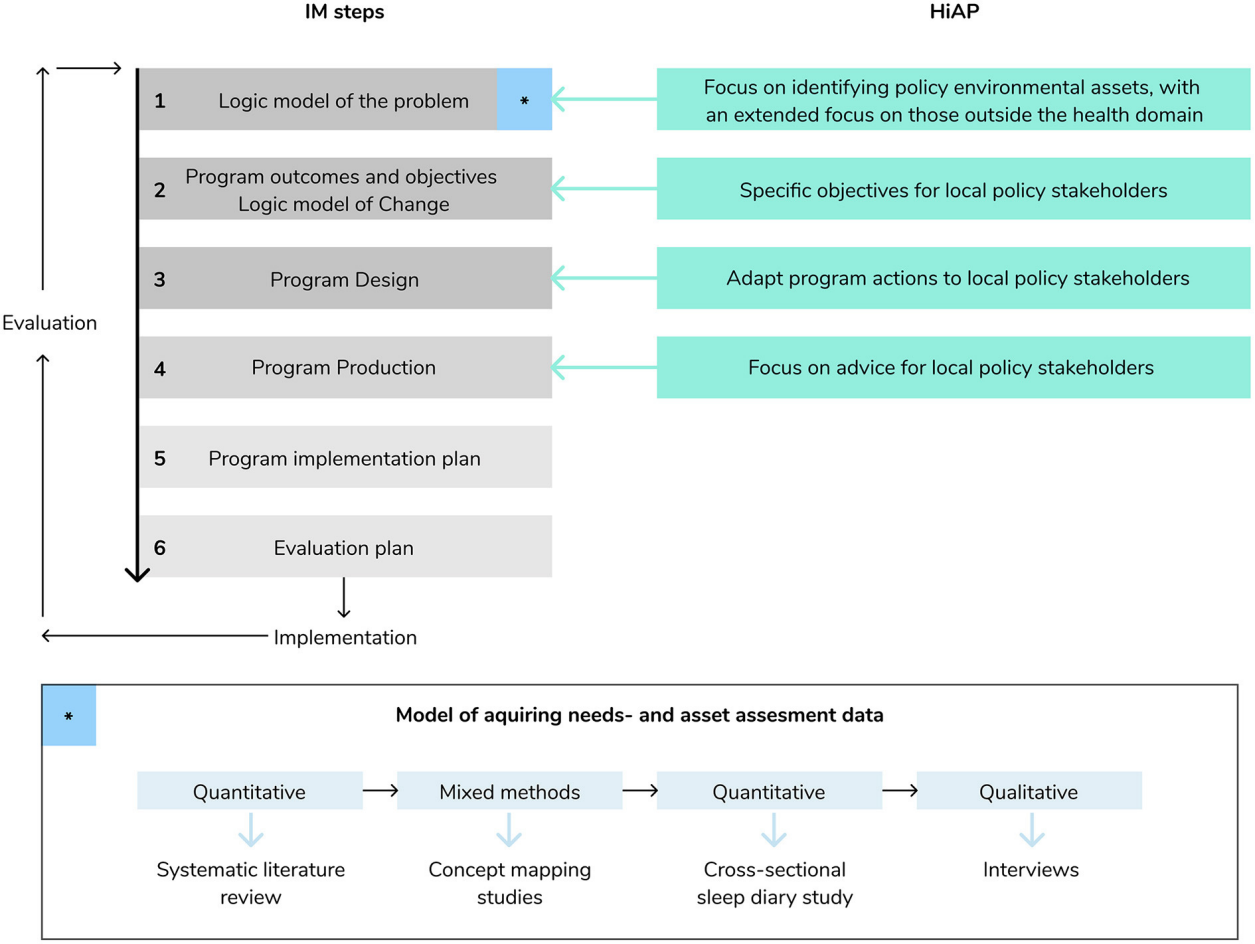
Development process combining IM with HiAP.

IM is a six-step protocol including (1) logic model of the problem; (2) program outcomes and objectives, logic model of change; (3) program design; (4) program production; (5) program implementation plan; and (6) evaluation plan. This paper describes our approach to the first four steps of the IM protocol. Below we explain step-by-step how the combination of IM and HiAP was applied for these four steps of IM. Figure 1 describes this development process.

### Step 1. Logic model of the problem

Creating a logic model of the problem produces a schematic overview of the factors related to a specific health problem, in this case, children's inadequate sleep health within the local context of the City of Amsterdam, which includes a population with various cultural backgrounds. This model is used to define the health problem, and to identify the ecological levels (e.g., interpersonal, community level) and related stakeholders connected to the problem. Creating a logic model of the problem consists of four tasks: (1) creating planning groups; (2) conducting a needs assessment, (3) describing the local context for the program, and (4) stating program goals. Regarding the first tasks, one of the planning groups as part of this project was an *advisory panel*, which included academic researchers with expertise in applying IM and developing preventative public health programs, sleep researchers, a somnologist, youth policy- and youth health advisors, a communication professional from the City of Amsterdam, and a policy manager. An advisory panel meeting was held two times on location, followed by individual consultation sessions with advisors separately. In addition to this advisory panel, we consulted a *parent advisory panel* within the community (*n* = 4–7) two times during this project. In addition, we consulted a group of parents working at the Public Health Service (*n* = 7) two times to test materials that were used in the parent advisory panel (e.g., a timeline exercise where parents mapped behaviors before bedtime) and to assist with creating performance- and change objectives.

The *needs assessment* (IM) included a combination of acquiring qualitative, quantitative, and mixed-methods data (see Figure 1). We conducted a systematic review to summarize the longitudinal evidence for factors related to children's sleep health (i.e., sleep duration, sleep quality, and sleep timing) (20). Next, two concept mapping studies were performed with different stakeholder groups (i.e., children aged 9-12 years, parents of children aged 4-12 years, and professionals) from various cultural backgrounds to explore the perceived determinants of children's inadequate sleep health (21, 22). These mixed-method studies were supplemented with a cross-sectional study among a culturally diverse group of children and their parents in Amsterdam (*n* = 382) to explore potential factors related to children's sleep (i.e., sleep duration, sleep quality, and sleep timing). A detailed description of the results of these studies are placed outside the scope of this paper and can be found elsewhere (20–23).

To get insight into the local context (i.e., local resources, capacities, and policy infrastructures) where the program will be implemented, and potentially existing local programs or program elements, an *asset assessment* was conducted (IM and HiAP) (12, 24). For this assessment, we used the framework created by Springer and Evans (24). This framework starts with identifying the settings where children and parents can be reached (e.g., neighborhoods, schools). When the settings had been mapped, we explored the environmental assets within those settings as part of four domains: (1) social environment; (2) information environment; (3) policy/practice environment (including the HiAP perspective); and (4) physical environment. To retrieve information on potential assets, we held 64 semi-structured interviews, between January 2019 and September 2020, with stakeholders and professionals in the field of policy, practice, and science. Local policy sectors were considered relevant when they were related to one or more behavioral- or environmental factors underlying children's inadequate sleep health. Additionally, HiAP created a focus on public administration and organization during these interviews. This directed the focus toward roles, assignments, and responsibilities of the different stakeholders. When the logic model of the problem was completed with the information from the needs assessment and the asset assessment, the program goals were specified.

### Step 2. Logic model of change

Creating a logic model of change produces a schematic overview of expected program effects and what is required to promote children's sleep health. Creating a logic model of change (IM) consists of five tasks: (1) stating expected behavioral- and environmental outcomes, (2) specifying performance objectives, (3) selecting personal determinants, (4) specifying change objectives, and (5) combining this in one structured schematic overview. The factors related to children's sleep health were used to determine the behavioral and environmental outcomes. Behavioral outcomes are behaviors that need to change as a result of the program at the level of the children; e.g., “children engage in a relaxing bedtime routine”. Environmental outcomes are environmental conditions that need to change in stakeholders at the interpersonal (e.g., parents), organizational (e.g., community and welfare organizations, parent- and child teams social services), community (e.g., community leaders, religious leaders, community center staff members), and society/policy levels (i.e., policy officers working for the relevant policy sectors). These outcomes were then further specified into performance objectives to provide a clear and exact description of the desired actions of stakeholders at the individual (i.e., children) and higher social-ecological levels (e.g., parents, child healthcare professionals, teachers, policy makers) to reach the outcomes; e.g., “children create a relaxing bedtime routine together with their parents”. To create change objectives (i.e., what needs to change related to the determinant to reach the performance objective), the performance objectives were combined with selected personal determinants. Personal determinants are factors that exist within individuals (children and other stakeholders) and can be changed or influenced by public health programs, such as knowledge of sleep promoting practices, attitude toward these practices, and the confidence in one's own ability to obtain such practices (i.e., self-efficacy). Examples of change objectives linked to this performance objective are “parents list the reasons for creating a relaxing bedtime routine” (knowledge) and “parents express positive feelings toward a relaxing bedtime routine” (attitude). Supplementary Table S1 presents an explanation for the selection of personal determinants of parents, which was based on our previous studies (20–23), other relevant literature (25–33), and theoretical models for health behavior (34–37). To select the personal determinants of children, the research team used the list of parental personal determinants and checked these on their relevance for children. The personal determinants were only excluded for children when this determinant was perceived as inappropriate for that performance objective. Matrices of change objectives were created. For this study, we focused on specifying the behavioral outcomes for children (aged 4–12 years) and environmental outcomes for parents (based on the interpersonal level) and the related performance and change objectives. See Table 1 for more examples. For each of these objectives, theoretical methods need to be selected and incorporated in practical applications, i.e., the actual program elements. These program elements need to be carefully tailored to the local context and culture, and developed together with different stakeholder groups. The objectives for stakeholders outside the intrapersonal and interpersonal level were not included in the logic models, because the higher-level environmental stakeholders differ per setting, while objectives for children and parents are relatively stable compared to other stakeholders. However, some examples are presented for different multi-sector policy stakeholders in Table 2. These objectives are linked to HiAP maturity stages; (0) unrecognized (i.e. no specific attention for the problem), (1) recognized (i.e. recognition of the problem and HiAP solution), (2) considered (i.e. preparatory HiAP actions on part of the problem), (3) implemented (i.e. HiAP investments in multiple problem areas), (4) integrated (i.e. quality HiAP processes integrated as part of policy), and (5) institutionalized (i.e. systematic improvement of quality HiAP) (38).

**Table 1:** Examples of performance and change objectives for children and parents.

**Performance objective**	**Personal determinants**						
	**Knowledge**	**Awareness**	**Attitude**	**Self-efficacy**	**Skills**	**Perceived barriers**	**Subjective norm**
**Behavioral/environmental outcome: Children obtain a relaxing bedtime routine/parents hold a relaxing bedtime routine for their child**							
Children create a relaxing bedtime routine together with their parents	• List reasons for creating a relaxing bedtime routine • List relaxing activities	• Review current bedtime situation • Recognize the need to create a relaxing bedtime routine	Express positive feelings toward a relaxing bedtime routine	Express confidence in ability to create a relaxing bedtime routine with their parents			Experience that most children have a relaxing bedtime routine
Children adhere to the relaxing bedtime routine they created with their parents			Express positive feelings toward adhering to the relaxing bedtime routine	Express confidence in ability to adhere to the relaxing bedtime routine			
Parents talk to the other parent/caregiver about creating a relaxing bedtime routine for their child	Define a relaxing bedtime routine	Recognize the need to discuss their current bedtime situation	Express positive feelings about discussing a bedtime routine for their child, together with the other parent/caregiver	Express confidence in ability to talk to the other parent/caregiver about creating a relaxing bedtime routine for their child	Demonstrate ability to discuss with the other parent/caregiver about creating a relaxing bedtime routine for their child	Anticipate negative responses of other parent/caregiver toward creating a relaxing bedtime routine for their child	Explain that most parents/caregivers discuss creating a relaxing bedtime routine for their child
Parents create a relaxing bedtime routine together with their child	List reasons for creating a relaxing bedtime routine	Recognize the importance to create the bedtime routine together with their child	Express positive feelings toward a relaxing bedtime routine	Express confidence in ability to create a relaxing bedtime routine	Demonstrate ability to create a relaxing bedtime routine	Anticipate negative responses of their child about creating a relaxing bedtime routine	Experience that most parents/caregivers have incorporated a relaxing bedtime routine
Parents discuss the logic behind a relaxing bedtime routine with their child	Recall reasons for discussing the logic behind a new bedtime routine with their child	Recognize the need to discuss the logic behind a relaxing bedtime routine with their child		Express confidence in ability to discuss the logic behind a relaxing bedtime routine with their child	Demonstrate ability to discuss the logic behind a relaxing bedtime routine with their child		Explain that most parents/caregivers discuss the logic behind a relaxing bedtime routine with their child
Parents consistently adhere to the relaxing bedtime routine before taking him/her to bed	Recall their child's relaxing bedtime routine	Recognize the need to adhere to the relaxing bedtime routine of their child	Express positive feelings about consistently adhering to the relaxing bedtime routine before taking their child to bed	Express confidence in ability to carry out the relaxing bedtime routine with their child every day, regardless of the circumstances	Demonstrate ability to adhere to the created relaxing bedtime routine before taking their child to bed		

**Table 2:** Examples of performance and change objectives for policy stakeholders.

**Performance objective**	**Personal determinants**						
	**Knowledge**	**Awareness**	**Attitude**	**Self-efficacy**	**Skills**	**Perceived barriers**	**Subjective norm**
**Environmental outcome: Policy makers from various sectors incorporate children's sleep health and/or its underlying factors in their work**							
Child health care and youth policy makers incorporate a broadly shared vision on HiAP (political and strategic) for children's sleep health and its underlying factors	Recall the relevance of incorporating a broadly shared vision on HiAP for children's sleep health	Recognize the importance of incorporating a broadly shared vision on HiAP for children's sleep health	Express positive feelings toward incorporating a broadly shared vision on HiAP for children's sleep health and underlying factors	Express confidence in ability to incorporate a broadly shared vision on HiAP for children's sleep health		Anticipate negative responses of council members toward incorporate a broadly shared vision on HiAP for children's sleep health	Experience that most municipalities incorporate a broadly shared vision on HiAP for children's sleep health
Public health policy makers collaborate with other sectors to prioritize children's sleep health (i.e., stakeholder engagement)	Recall the relevance of collaborating with other sectors to prioritize children's sleep health	Recognize the importance of collaborating with other sectors to prioritize children's sleep health	Express positive feelings toward collaborating with other sectors to prioritize children's sleep health	Express confidence in ability to collaborate with other sectors to prioritize children's sleep health	Demonstrate ability to advocate the importance of collaborating with other sectors to prioritize children's sleep health	Anticipate negative responses of policy stakeholders outside the public health domain toward collaborating to prioritize children's sleep health	Experience that within most municipalities policy sectors collaborate to prioritize children's sleep health
Education policy makers politically and administratively anchor the HiAP approach for children's sleep health and its underlying factors (i.e., sustainable implementation)	Recall the relevance of politically and administratively anchoring the HiAP approach for children's sleep health	Recognize the importance of politically and administratively anchoring the HiAP approach for children's sleep health	Express positive feelings toward politically and administratively anchoring the HiAP approach for children's sleep health	Express confidence in ability to politically and administratively anchor the HiAP approach for children's sleep health			Experience that most municipalities politically and administratively anchor the HiAP approach for children's sleep health
Social security and welfare policy makers incorporate HiAP in their policy documents to promote children's sleep health and its underlying factors	Recall the relevance of incorporating HiAP in their policy documents to promote children's sleep health	Recognize the importance of incorporating HiAP in their policy documents to promote children's sleep health	Express positive feelings toward incorporating HiAP in their policy documents to promote children's sleep health	Express confidence in ability to incorporate HiAP in their policy documents to promote children's sleep health			Experience that most municipalities incorporate HiAP in their policy documents to promote children's sleep health

### Step 3. Program design

Designing the program includes selecting suitable change methods for behavioral- and environmental outcomes, and potential practical program elements. From the IM point of view, our aim was to design a coherent, deliverable program. The HiAP perspective contributes to the creation of a program that can be structurally embedded in existing organizational structures of public policy and practice. Consequently, we first mapped the existing organizational structure of the local policy environment. Next, we followed an iterative process wherein we selected potential practical applications *via* a context-driven HiAP approach. This was followed by an exploration of potential practical applications, mechanisms, and communication channels based on (1) feasibility, (2) usefulness, and (3) extent of implementation in existing policy and practice structures. For this, we used semi-structured interviews with different stakeholders, such as policy professionals, child healthcare professionals, and community management professionals. The exploration of potential practical applications was an iterative process, where every interview could lead to new potential applications and new stakeholders for an interview. This resulted in a set of program elements that fits this structure and therefore promotes successful implementation. Furthermore, to build upon what already exists in Amsterdam, we created an overview of existing practical applications and services for the behavioral-, environmental-, and personal factors of children's sleep health. Based on what already existed and the interviews with stakeholders, we created a set of potential practical applications that fit the local environmental structure and therefore promote successful implementation. This final set of program elements can be constituted as one multi-sector approach to promote children's sleep health within the local context of Amsterdam.

### Step 4. Program production

Based on the program design, together with professionals from policy and practice, we created an extensive policy brief for the City of Amsterdam's aldermen and policy officers working for the policy sectors related to children's sleep and its underlying factors. During the design of this policy brief, continuous interaction between the research team, the teams of “The Amsterdam Healthy Weight Approach”, “Sarphati Amsterdam” research institute, and policy makers, resulted in opportunities per relevant policy sector on how they can increase their impact on the behavioral- and environmental factors related to children's sleep health. For each policy sector, at least one policy officer was asked to collaborate in creating the policy brief. This policy brief aimed to promote the HiAP process from stage 0 “unrecognized”, to stage 1 “recognized”. The policy brief will help the aldermen and policy officers to recognize the problem and the solution of HiAP and offer a perspective on how to organize an effective approach to alleviate the problem (38).

## Results

This section presents a stepwise description of the findings of the first four steps of IM.

### Step 1. Logic model of the problem

**Figure 2:**
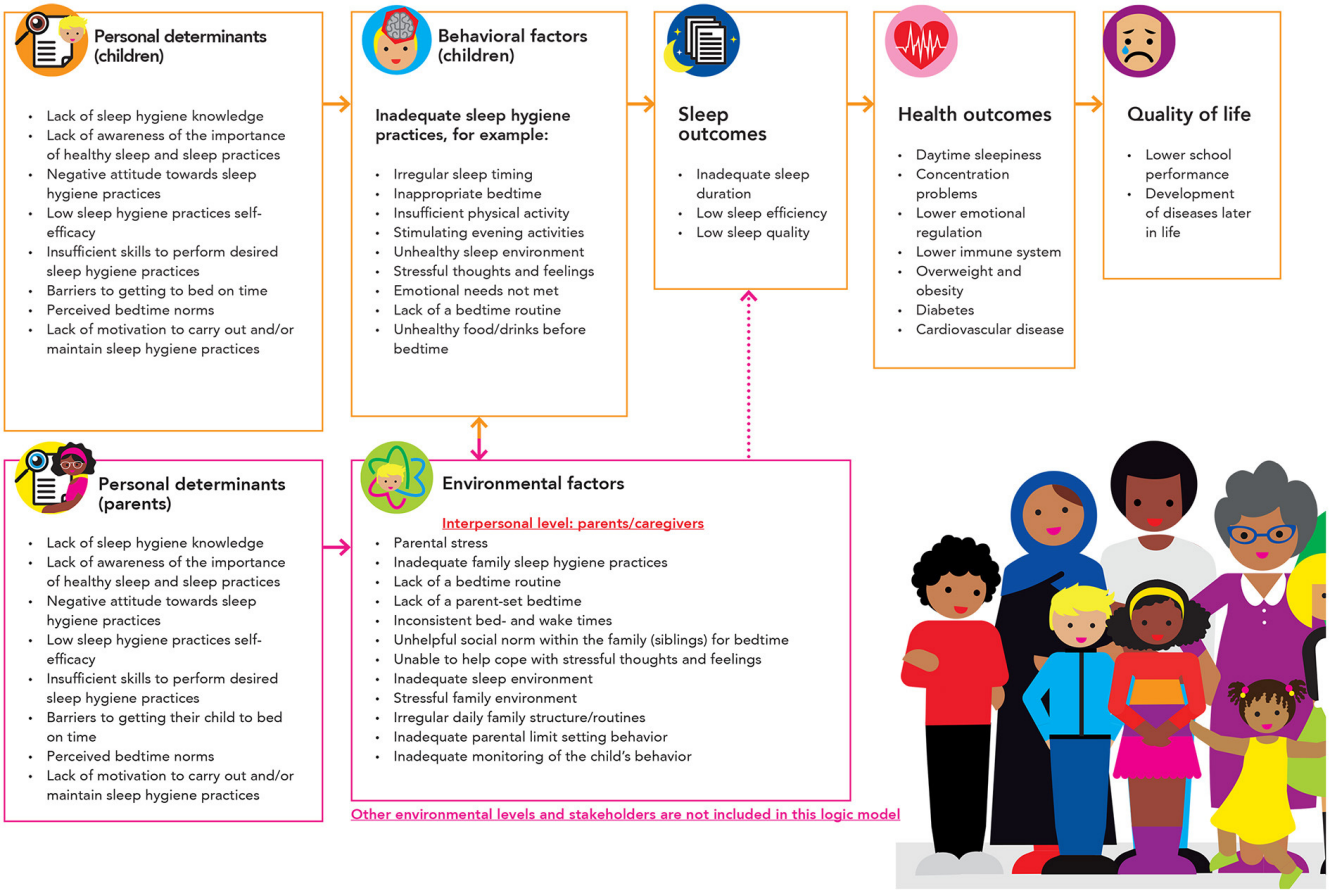
Logic model of the problem.

We created a logic model of the problem (Figure 2). This model gives an overview of the identified quality of life outcomes, health outcomes, sleep outcomes, behavioral factors (on the interpersonal level; children), environmental factors (on the intrapersonal level; parents), and the personal determinants related to these factors. Most behavioral factors were related to sleep promoting practices, which can be defined as “a set of recommended behavioral- and environmental practices intended to promote healthy sleep” (39). Selected sleep promoting practices for this context included irregular sleep timing (i.e. bedtime variability), inadequate bedtime, inadequate amount of daytime physical activity, an unhealthy sleep environment (i.e. inadequate temperature, light or noise, uncomfortable bed), inability to let go of fear and worries and to relax, no bedtime routine, active activities close to bedtime (e.g. screen use, active games), emotional needs were not met, eating large amounts of food or products with a high amount of sugar or caffeine close to bedtime, inadequate exposure to daytime light, and the inability to fall asleep independently. The environmental factors on the level of parents included parental stress, poor sleep practices, inability to provide emotional support, and inadequate parenting practices (e.g., inability to set rules, monitor child's behavior, and create daily family routines). Both behavioral- and environmental factors relate to personal determinants. We identified eight personal determinants in total.

**Figure 3:**
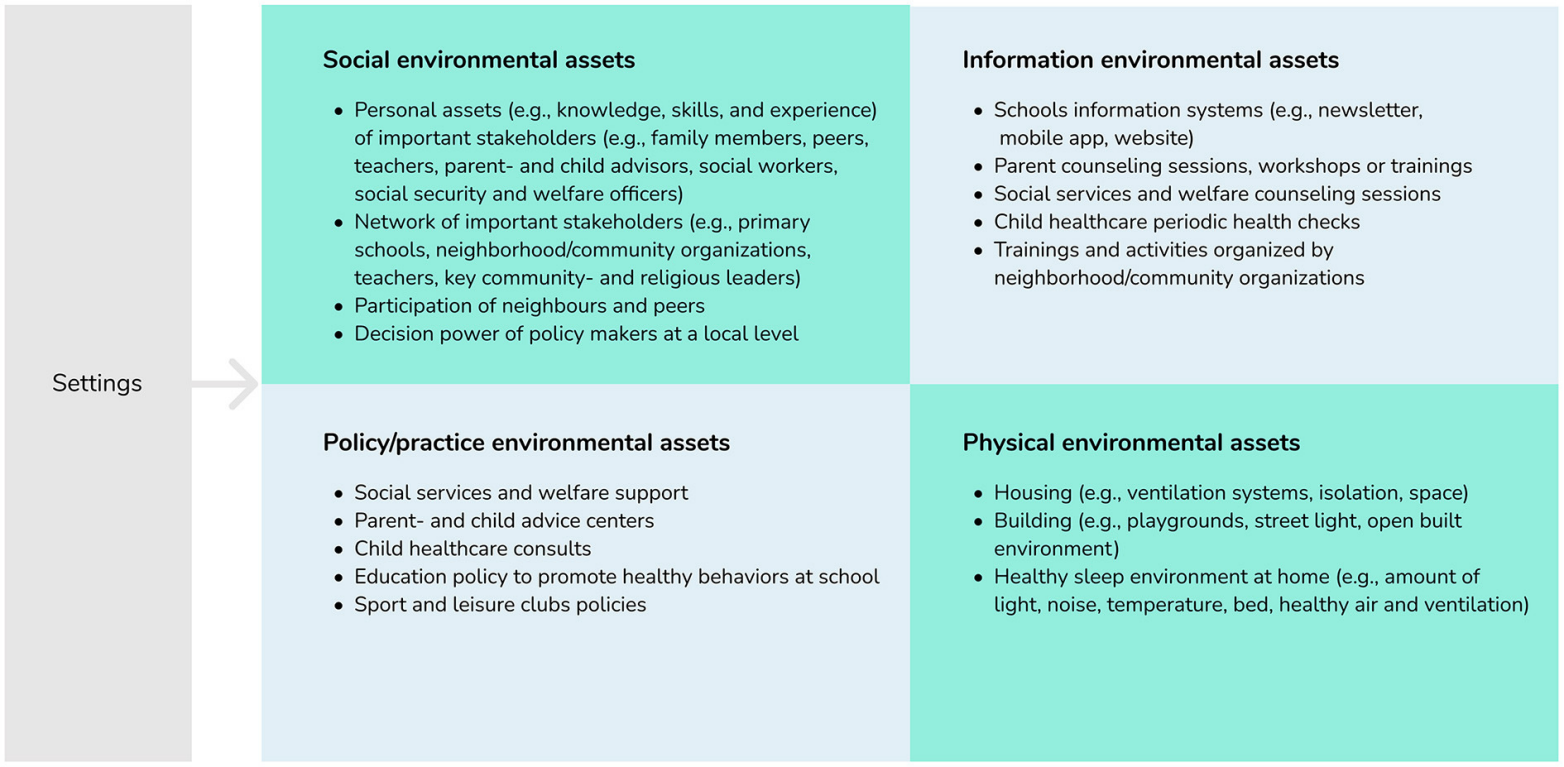
Environmental asset assessment, examples per domain.

The logic model of the problem was complemented with an asset assessment (12, 24). Figure 3 illustrates some of the environmental assets for each of the four domains: social environment, information environment, policy/practice environment, and physical environment. The social environment included for example all personal assets of the important stakeholders and their network. The information environment included several types of information structures that could be used to reach the target population with a message *via* an existing communication channel. The policy/practice environment included the policies and practices, and existing local programs or program elements that can be exploited or improved to positively stimulate the underlying factors of children's inadequate sleep health. For example, social services and welfare support by the municipality already support families that are not yet financially independent, and this structure could be used to explore ways of providing further support, as financial instability creates a stressful family situation. The physical environment includes features of the built environment that could support children's adequate sleep health, such as housing and building. To select the program context, we integrated the HiAP perspective by including all relevant policy and practice assets for potential inclusion within the multi-sector program.

Based on the needs- and asset assessment, three program outcomes were set: (1) Children have healthy sleep practices; (2) Parents have healthy family sleep practices; (3) Multi-sectoral environmental assets support children and parents in healthy sleep practices, e.g., policy makers from various sectors incorporate children's sleep health and/or its underlying factors in their work.

### Step 2. Logic model of change

**Figure 4:**
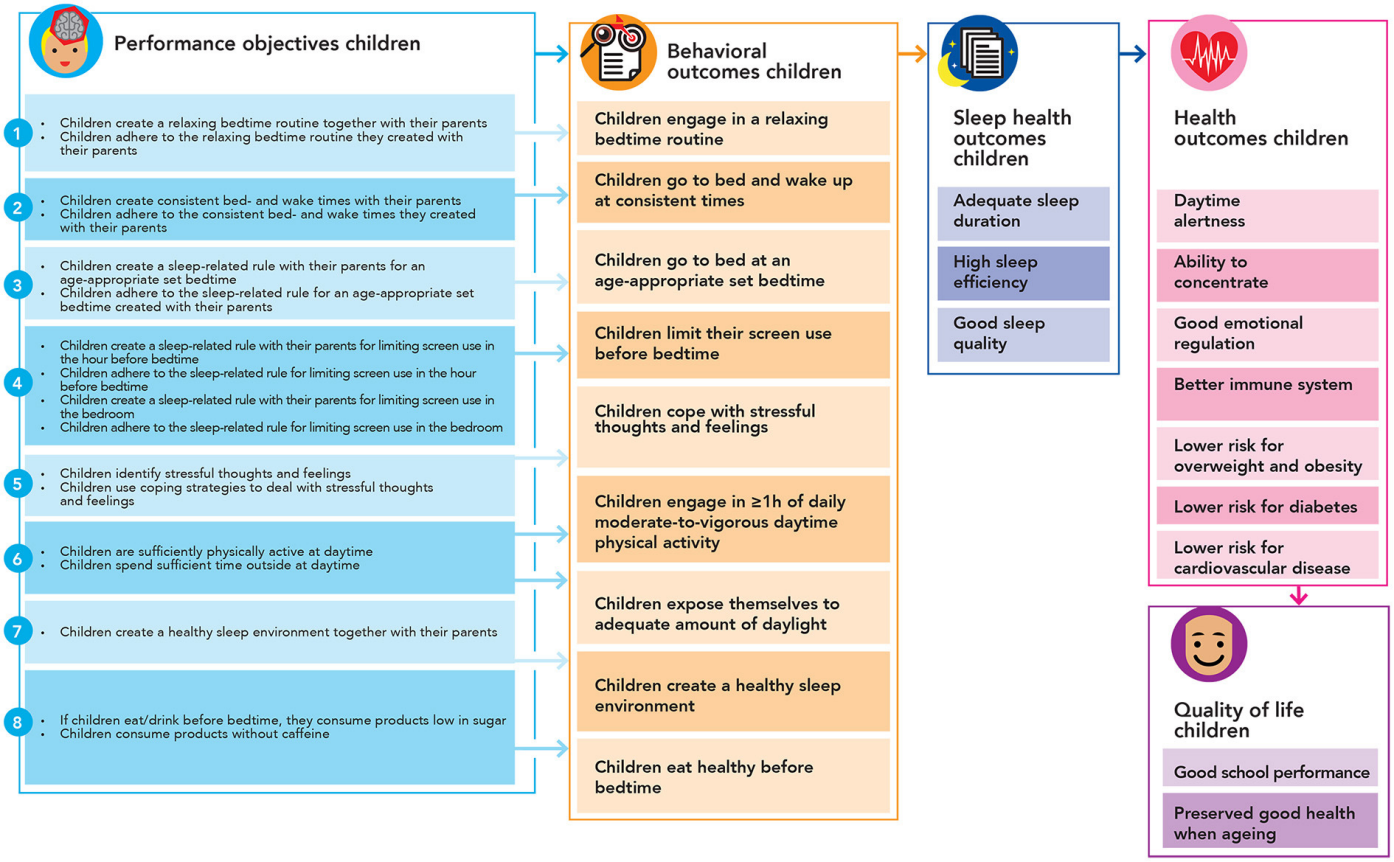
Logic model of change for children.

**Figure 5:**
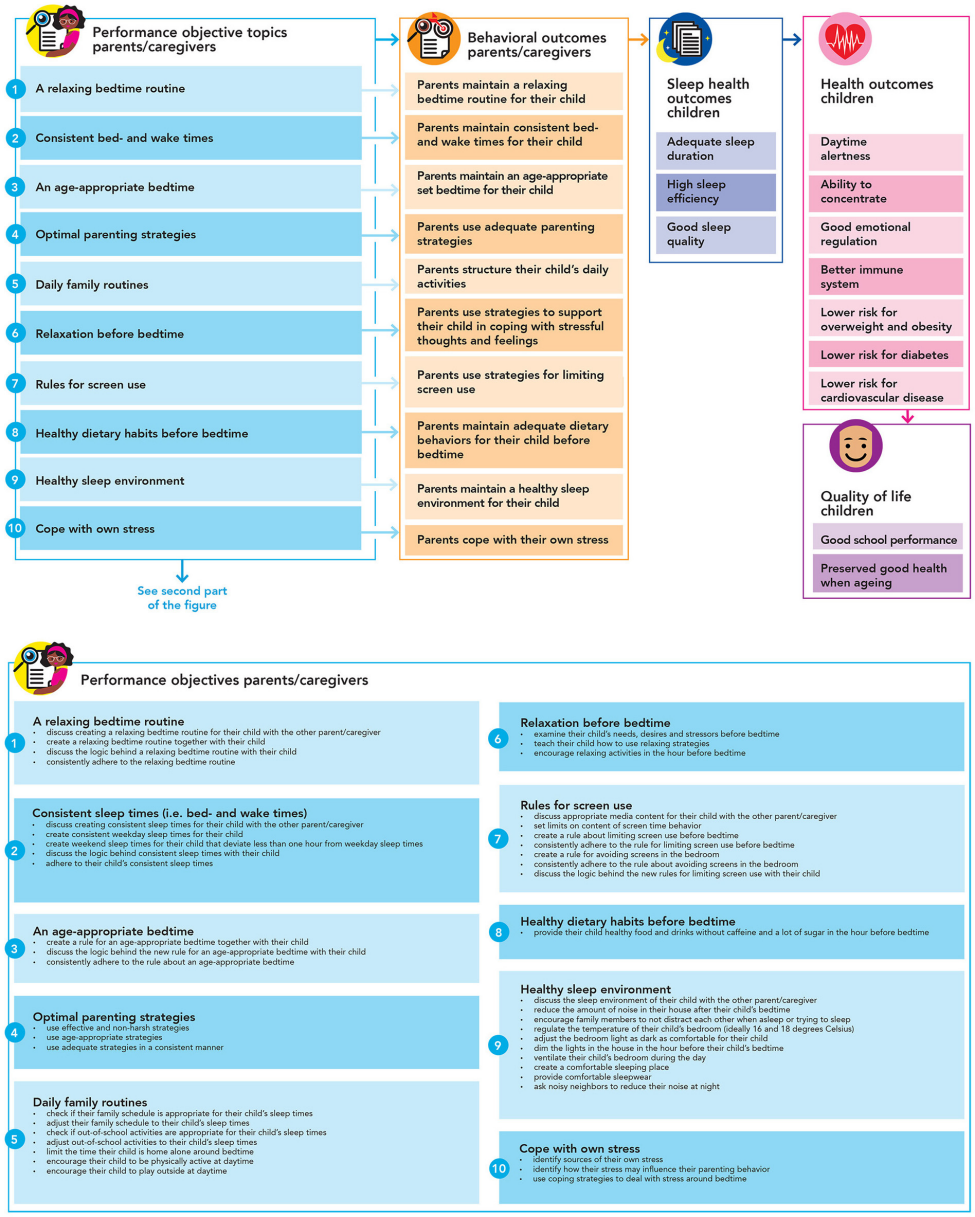
Logic model of change for parents.

We created a *logic model of change* to specify what performance objectives needed to be addressed to succeed in realizing the behavioral- environmental-, and program outcomes. This logic model of change was created for children (see Figure 4) and parents (see Figure 5). These logic models imply how a potential change in the personal determinants (e.g., knowledge, attitude, skills) is expected to impact the specified behavioral outcomes (e.g., healthy sleep practices) in children and parents. These change models also show how, in turn, more distal outcomes (i.e., sleep health and quality of life) are consequently impacted. These models of change are illustrative, not exhaustive, meaning that other changes besides parents' and children's personal determinants should be addressed eventually, such as higher-level environmental factors (e.g., characteristics of the built environment) and personal determinants of other stakeholders, e.g., policy makers, child healthcare professionals, and teachers. These logic models of change provide an overview of what factors need to be addressed *via* a health promoting approach within this local context. In a different context, these factors might be different according to the local context and culture. However, it is still unclear how these changes are to be realized. To clarify this, we applied IM to map what outcomes need to change, to create a whole of required changes and actions that together will make up the program. Tables 1, 2 present several illustrations of such created performance and change objectives. These objectives are not the program's messages and are only used by the program developers to match with appropriate evidence-based theoretical methods, and translate these methods into program elements including culturally sensitive messages.

### Step 3. Program design

**Figure 6:**
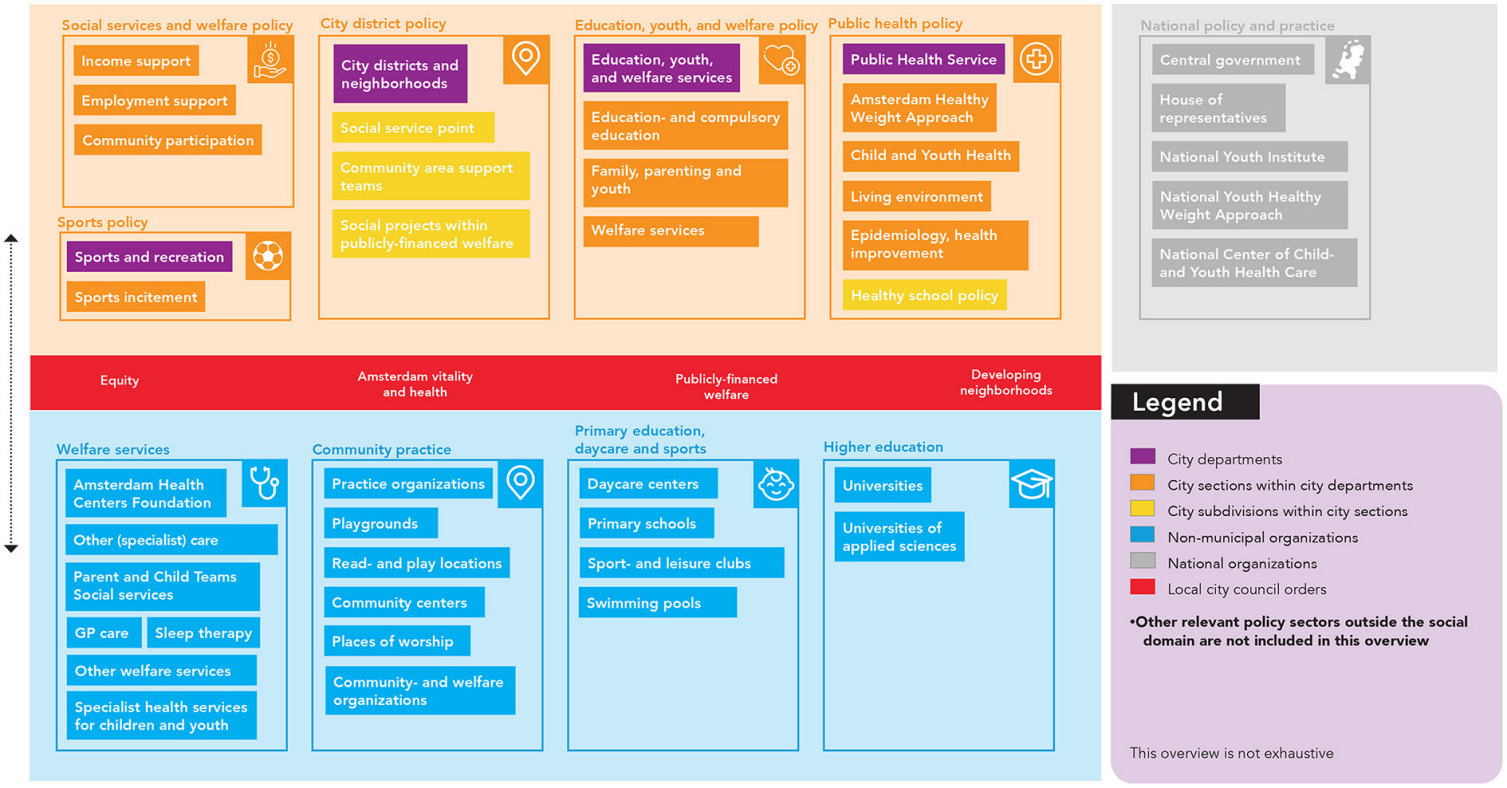
Overview of the local organizational system.

Creating the program goals, performance- and change objectives mapped the changes required to effectively impact children's sleep health. The next step in IM was to match these change objectives to appropriate theoretical methods for behavioral- or environmental change. However, to create a program that can be implemented in a durable and feasible way, these methods do not only need to be effective tools to change certain determinants, but also fit one's specific context of use. To ensure this match, we first carefully mapped Amsterdam's policy and professional practice infrastructures related to influencing (underlying factors of) children's sleep health (Figure 6). We displayed the organizational policy structure, the organizational practice structure, relevant local city council orders, and national policies and organizations that are related to one or more organizational structures for policy and practice at the local level. We note that this overview is not exhaustive.

Table 3 presents the opportunities for sleep health in all policies for each identified public policy sector. We included all policy sectors within the social domain and an exploration of policy sectors outside the social domain. One example of an opportunity is that the education policy sector could incorporate sleep health and its underlying factors in education programs focused on promoting children's health. By promoting children's sleep health, they also promote education equality as well-sleeping children are better able to concentrate and perform within the education system.

**Table 3:** Opportunities for sleep health in all policies for local policy in Amsterdam.

**Policy sector**	**Description of policy sector**	**Opportunities for promoting children's sleep health**
Health care and youth policy	Health care and youth policy strives to provide quality care for vulnerable households.	Health care and youth policy could influence the underlying factors of children's inadequate sleep, e.g., stressful family situation, parenting skills, children's fear and worries, parental stress.
Education policy	Education policy strives to provide children with quality education and minimize school absenteeism.	Health education programs could address sleep health and its underlying factors. Children's healthy sleep also promotes equity in education, as they are better able to concentrate and perform at school.
Sport policy	Sport policy strives to create a diverse range of sports- and exercise activities, stimulating sports and exercise and creating an active-friendly environment.	By promoting physical activity, sport policy indirectly already contributes to healthy sleep practices, as one of the sleep promoting practices is adequate physical activity. Children's healthy sleep is also important for children's health and energy level and could promote sports performance.
Public health policy	Public health policy supports the promotion of citizen health and reducing health disparities.	Positively stimulating sleep health and its underlying factors fits well within this policy, as this sector impacts factors that influence children's sleep, such as helping parents deal with stress or with developing parenting skills.
Social services-, welfare and poverty policy	Social services and welfare policy supports the guidance of welfare recipients toward work or participation. Poverty policy combats poverty with active and optimal income support.	These policies could indirectly impact the underlying factors of children's inadequate sleep health (e.g., stressful family situation, unhealthy sleep environment). Children's healthy sleep could also reduce parental stress.
Community participation policy	Community participation policy strives to enlarge participation of citizens through community activities.	Community activities could include sleep health and its underlying factors and are part of the organizational structure for transmission of (sleep) health information.

Other policies outside the social domain that can be explored are: spatial development policy, community services policy, mobility and public space policy, and national policies. In addition, partnerships with human services organizations or partly-financed governmental organizations can be explored.

Based on the results of the needs- and asset assessment, opportunities for program actions were created in close collaboration with policy- and practice stakeholders. Supplementary Table S2 presents these program actions linked to the policies presented in Figure 6. The program design was put into a physical end-product: a local policy brief. This document describes the opportunities and program actions for each policy sector. The target audience for this policy document are the aldermen and all policy officers at the City of Amsterdam, working in one of the included policy sectors.

### Step 4. Program production

The product we created was the local policy brief. This policy brief creates awareness of the problem, the opportunities of HiAP as a solution to this problem, and clarity on potential program actions for each policy sector. *Via* the alderman and policy officers, the policy brief promotes the incorporation of children's sleep health and its underlying factors in multiple policy sectors in the city. The brief states the importance of children's sleep health related to each specific policy sector, sets out potential program actions for each sector, and describes how these sectors can effectively operationalize sleep health in their policy. The policy brief includes several infographics to increase comprehensibility. The full policy brief is in Dutch, not publicly available and therefore not included in this paper. The owner of this policy brief is the public health sector within the City of Amsterdam, and they are the responsible organization for advocating this policy brief and its implications within their organization. The policy brief was finalized and shared with the City of Amsterdam in February 2021. The policy makers responded positively to the proposed approach and potential health promoting actions. In the Amsterdam Health Policy brief (period 2021–2025) (40), that received unanimous support of the Amsterdam City Council in December 2021, the topic of sleep health was adopted. The Amsterdam Health Policy is a joined policy brief that affects all parts of the local administration, which offers opportunities to realize the recommendations of the policy brief focused on children's sleep health.

## Discussion

This paper describes a blueprint for the development of a program to promote children's sleep health by combining Intervention Mapping (IM) with Health in All Policies (HiAP). This resulted in a systematically developed outline to produce and structurally implement an integrated approach to promote children's sleep health across multiple policy domains, and a local policy brief.

As children's sleep health is influenced by a broad scope of various interconnected individual, social, and environmental factors (20), there is a need for a multi-level approach. IM provides a methodology to design such an approach in an evidence-based way (12). Combining IM with HiAP adds a focus on identifying the appropriate and relevant public policy sectors, and policy assets (i.e. existing local policy-related resources and capacities) to create an integrated, city-wide approach (15, 41). Many public policy sectors, (e.g. social security, education) influence (underlying factors of) children's sleep health (16, 41). By additionally including relevant non-public health policy sectors *via* HiAP, program outreach can be increased significantly (42). This combination of program development methodologies (i.e., IM with HiAP) could also benefit other health-behavior programs. Our approach takes the local (policy) context and culture into account and provides the most important stakeholders with tools to start changing factors related to (sleep) health behavior. The blueprint for program development that we created in this study is not restricted to the Amsterdam context but can be adapted to fit any local (policy) context, culture, and set of stakeholders.

One of the lessons learned while applying HiAP in our sleep health context was the importance of finding a joint interest within each policy sector that is related to the problem and use this common ground to build partnerships. Often, policy sectors other than the health sector are indirectly related to children's sleep *via* underlying factors, such as a stressful family situation or housing. However, in general, they did not view children's sleep health as a shared responsibility. As previous research showed, working from such a win-win starting point created stronger partnerships and more valuable results (43). To provide an example, many underlying factors of children's sleep health disproportionately affect families with a low socioeconomic position (44, 45). Since many of Amsterdam's municipal policy sectors are (partly) responsible for tackling the complex problem of (health) inequity, there could be a great deal of common ground. This can serve as a broad basis of support to engage policy makers from several policy sectors to create shared ownership in tackling the issue of children's inadequate sleep health. This shared ownership can further be stimulated by involving policy stakeholders early on in the research- and development process and creating co-ownership of program actions (46). The HiAP Maturity Model (38) incorporates several characteristics that underpin the importance of shared ownership, including the broadly shared vision on HiAP (political active engagement of governmental counselors and strategic inclusion of HiAP themes by the City Council), collaboration between sectors within the project (i.e. stakeholder engagement) and the political and administrative anchoring of the HiAP approach (i.e. sustainable implementation) within the governmental organization. Furthermore, this model enables the governmental organization to manage and control the HiAP processes (i.e., the key HiAP characteristics the organization tends to achieve), track their progress, and identify opportunities for improvement. In addition, the HiAP perspective created a specific focus during the interviews; on roles and responsibilities of stakeholders within the local organizational structure of policy and practice. This led to in-depth information on potential assets, which promotes a program design that fits the local context.

The blueprint for multi-sector program development created in the current study requires long-term involvement and commitment from both academia and partners from municipal policy organizations. This means commitment and involvement *via* e.g., stable funding mechanisms, establishing a supportive governmental structure with a shared HiAP vision across sectors, *via* creating ownership and shared responsibility for the HiAP implementation on the local level, and *via* engaging all sectors early on in the policy development (47, 48). To establish such commitment across sectors and allow policy stakeholders to give priority to HiAP actions, HiAP needs to be embedded in the local city council order. The HiAP progress can be monitored using the HiAP Maturity Model, which describes five levels of HiAP maturity and the characteristics for each level (38, 49). The development process toward an integrated approach to promote children's sleep health helped the city of Amsterdam to recognize both the problem and the importance of integral policy action; i.e., during the study period, Amsterdam moved from “Stage 0, unrecognized” to “Stage 1, recognized” within the HiAP Maturity Model in context of children's sleep health. Thereafter, it progressed to “Stage 2, considered”, since the public health sector made a policy statement that a HiAP approach is desirable when tackling the problem of children's sleep health. A future step toward further progress could be to create cross-sector collaboration and infrastructures. This could happen *via* a “HiAP Unit”, which Baum et al. defined as “A dedicated pool of skilled staff that could provide assistance across government, and was largely responsible for creating and maintaining a networked, horizontal governance for HiAP across”. Such a unit has shown to be helpful in gaining support from other sectors (38, 41, 50, 51). Another way might be to appoint one key policy sector (e.g., public health sector) as owner and main responsible sector for integrating HiAP within the local government, given sufficiently broad support among the other sectors. This owner can guide existing policy departments and their teams toward incorporating the HiAP perspective into their work and implement sustainable health-promoting policies. The decision on how to proceed depends on the local policy culture and context. Aiming to apply HiAP to create such structural impact within the governmental organization may also promote structural impact at different levels within the system of factors that influence children's sleep health. In case of sleep health specifically, it would also spill over to other social- and health related benefits, since sleep health is so strongly interwoven with other socially relevant outcomes and healthy child development (52).

Another lesson learned from applying HiAP was that valuable information about policy assets can be discovered by finding and involving stakeholders at all different hierarchical levels within the governmental civil service organization. Ideally, when developing public health programs, the direct implementers of the program are involved. These implementers are part of organizational sub-systems within the local government, which all work to realize their specific political goals and all have their standard ways of working. In our study it proved vital to first become thoroughly acquainted with the context of policy and practice, before being able to identify what specific stakeholders needed to be involved, which parts of their work practices required change, and where the power to realize those changes could be found. Furthermore, stakeholders at different hierarchical organizational levels can provide valuable information on policy assets. For example, they can help to understand how policies are operationalized in practice, how political agendas are shaped in local policies, and to provide knowledge on existing policies and governmental structures. Within our project, different policy stakeholders showed interest in cross-sector collaboration to promote the health of citizens. Collaborations created with and between such stakeholders are vital to sufficiently understand the system they aim to change together.

Although IM has long been a golden standard in behavior change intervention design, certain aspects appeared particularly important when designing a program blueprint for such a complex, multi-sector issue as children's sleep health. Firstly, incorporating a thorough needs- and asset assessment enabled us to ensure the developed actions would fit both community needs and existing environmental assets (12). In addition, the asset assessment helped to match existing resources and capacities in the communities to the actions that could be developed. This seems crucial for facilitating optimal program adoption, implementation and maintenance, which in turn is needed to create effective, sustainable programs (12). Secondly, we used a non-linear and iterative process of applying IM (12), as we went back and forth through the different steps in the IM process when new information appeared. This helped us to capture all necessary aspects of the system in which we aimed to create changes in order to stimulate children's sleep health.

Children's sleep health is shaped by the cultural context children live in, i.e. when, where, how, how much, and with whom children and families sleep (53). The program development approach that we described in this paper is suitable for developing programs in cross-cultural contexts, as the development approach (IM combined with HiAP) can be applied across various contexts and cultures (12). Additionally, HiAP specifically aims to create health equity, i.e. disproportionately promote the health of those who are most in need to close the gap between advantaged and disadvantaged groups through combined efforts of the relevant policy sectors (41). As in every health promoting program, specific attention needs to be directed to creating a culturally sensitive program that fits with the lived realities of the people that are at the center of the health topic at hand (54). In our project, we included stakeholders from different cultural backgrounds living in Amsterdam within the needs assessment (IM step 1). As a result, the program objectives are based on the local perspective of healthy sleep practices such as limiting the amount of noise, and sleeping alone. Instead of implementing such a locally informed program to another context, we propose to apply the blueprint for program development in another cultural context to promote cultural sensitivity of health promotion programs, and contributing to health equity (55, 56). Involving local cultural advisors throughout the project would be another way to improve cultural sensitivity of health promotion programs.

### Strengths and limitations

A strength of our study is the combination of IM and HiAP, which resulted in a blueprint for developing an integrated multi-sector program to promote children's sleep health. Furthermore, using participatory research methods with children, parents and other stakeholders ensured the program was optimally geared toward the priority stakeholders. Another strength was that this research was performed through close, structural collaboration between academia, public policy, and public health practice. However, there are also some limitations to our approach. The needs assessment could have been extended by identifying the most relevant personal determinants quantitatively e.g., on basis of the confidence interval based estimation of relevance (CIBER) approach instead of theoretical models and supporting research (57, 58). However, this was not possible because the personal determinants in our study were not specified per behavioral factor (57, 58). Furthermore, we did not include performance- and change objectives for *all* environmental stakeholders, which would have created an even broader socio-ecological approach to influence children's sleep health. However, since relevant environmental and policy structures differ per region, we want to encourage policy makers to identify performance and change objectives for their own sector when implementing the blueprint for program development that we offer *via* this paper.

## Conclusion

Combining IM with HiAP for health promotion resulted in a comprehensive, evidence-based blueprint for the development of an integrated multi-sector program to promote children's sleep health. This blueprint can also serve to support the design of local (sleep) health promotion programs in other places with different local governmental structures within different cultures while taking into account the policy context.

## Data availability statement

The raw data supporting the conclusions of this article will be made available by the authors upon reasonable request, meaning that they may be shared with researchers who provide a methodologically sound proposal and whose proposed use of the data has been approved by the study's authors and partners.

## Ethics statement

The studies involving human participants were reviewed and approved by Medical Ethics Committee of the VU University Medical Center (Protocol No. 2017.013 and 2018.170). Written informed consent to participate in this study was provided by the participants and/or one of the participants' parent or caregiver where appropriate.

## Author contributions

LB, MVS, IH, KDH, RR, MC, and VB conceptualized and designed the research project. LB, MVS, KDH, RR, MC, and VB interpreted the data. LB collected the data and wrote the initial manuscript and all other authors contributed to writing and editing the manuscript. All authors have read and agreed to the published version of the manuscript.

## Funding

This research was funded by the Amsterdam Healthy Weight Approach, Public Health Service (GGD), City of Amsterdam, Amsterdam, the Netherlands, and Scientific Research Institute Sarphati Amsterdam, Public Health Service (GGD), City of Amsterdam, Amsterdam, the Netherlands.

## Conflict of interest

The authors declare that the research was conducted in the absence of any commercial or financial relationships that could be construed as a potential conflict of interest.

## Publisher's note

All claims expressed in this article are solely those of the authors and do not necessarily represent those of their affiliated organizations, or those of the publisher, the editors and the reviewers. Any product that may be evaluated in this article, or claim that may be made by its manufacturer, is not guaranteed or endorsed by the publisher.

## Abbreviations

HiAP: Health in All Policies
IM: Intervention Mapping.
