# Supplementary material for: Promoting children's sleep health: Intervention Mapping meets Health in All Policies
Source: Front Public Health. 2022 Nov 16;10:882384. doi: 10.3389/fpubh.2022.882384 (PMC9709501; doi:10.3389/fpubh.2022.882384)
Supplement: Supplementary file 1 [file Data_Sheet_1.pdf]

Supplementary material

Table S1. Explanation of the selection of personal determinants for parents/caregivers

**Personal determinants**	**Explanation**
Awareness of the importance of sleep health and the underlying factors	Parents may perceive their child as a healthy sleeper, even though their child might actually not sleep adequately (1). Furthermore, the assumption is that an individual will not change their behavior unless they are aware that change is important and necessary (i.e. precaution adoption process model) (2).
Knowledge about healthy sleep and sleep practices	Parents may lack knowledge about healthy sleep practices for children (3). This is also an important element in the integrated behavioral model (4).
Attitude towards children's sleep health	Parents may have dysfunctional beliefs about children's sleep (3, 5). This is also an important personal determinant in the integrated behavioral model (4) and the theory of planned behavior (6).
Parenting self-efficacy	Many of the underlying factors of children's inadequate sleep are related to parenting (5, 7-9). Parenting self-efficacy may be related to children's sleep (10, 11). Several health behavior models support the importance of this personal determinant, i.e., social cognitive theory (12), health belief model (13), integrated behavioral model (4), theory of planned behavior (6).
Parenting skills	Several underlying factors that were found, such as rule setting, daily family structure and routines, have to do with parenting skills (5, 7-9). This is also an important personal determinant in the integrated behavioral model (4).
Perceived barriers	Parents identified barriers that prevent them from making changes within their family routines and habits (8) and this was also found among professionals (7). Parents perceived a barrier to get their child to bed on time when siblings have a later bedtime (14). The health belief model (13) underpins the importance of this personal determinant.
Perceived social norms	There may be some perceived social norms that are relevant, such as other children who go to bed later (i.e., peers/siblings) (7, 8). The relevance of perceived social norms is substantiated in the integrated behavioral model (4) and the theory of planned behavior (6).
Motivation	It is unlikely that an individual will change their behavior without the motivation to change (i.e. integrated behavioral model) (4).

Table S2. Opportunities for program actions per policy sector

Policy sector	Opportunities for program actions
Health care and youth	• Include prevention focused on healthy sleep in health care policy and youth policy<br>• Focus on prevention of children’s inadequate sleep by child health care and parenting advice professionals<br>• Educate child health care and parenting advice professionals on healthy sleep and its underlying factors<br>• Promotion of healthy sleep, by child health care and parenting advice professionals, through signaling underlying problems<br>• Include information about healthy sleep in the child health care communication channels<br>• Strengthen the connection between parents and child health care and parenting advice professionals<br>• Adjust training programs of parenting advice teams to the needs of parents<br>• Use children’s yearly health information to improve the service of child health care and parenting advice teams<br>• Optimize the use of the electronic children’s health care program<br>• Develop standardized thematic parenting sessions about children’s sleep health<br>• Healthy sleep curriculum for primary schools<br>• Healthy sleep integrated in healthy school policy
Public health	• Valid and reliable monitoring of children’s sleep and its underlying factors<br>• Agenda setting for a healthy sleep environment for public policy<br>• Agenda setting and embedding of healthy sleep within relevant trainings and courses for future professionals
Education	• Include healthy sleep and its underlying factors in education policy<br>• Explicit attention in absenteeism policy for children’s inadequate sleep and the signaling role of the compulsory education officer<br>• Explore the role of pre-school and children’s daycare organizations (e.g., role model for healthy sleep practices)
Community participation	• Optimize connection between the community and child health care and parenting advice professionals<br>• Integrate healthy sleep within existing community activities<br>• Set conditions that promote healthy sleep for subsidized community activities

Sports	• Structurally integrate healthy sleep for all ages in a lifestyle program by a role model company (e.g., a well-known sports club)<br>• Structurally integrate healthy sleep in education for sports- and leisure professionals and activities of sports- and leisure clubs
Social security and welfare	• Include the focus on the underlying factors of children's inadequate sleep health in social security and welfare policy<br>• Explore whether client journeys can be optimized within existing social security and welfare services<br>• Broaden the knowledge and skills of social security and welfare officers<br>• Strengthen collaboration between social security and welfare professionals with other policy sectors<br>• Explore whether resources for low-income families are adequate and enable access to stress reduction programs and materials to create a healthy sleep environment at home
Spatial planning	• Integrate factors within urban planning policy to create sleep promoting environments
National	• Use role models to promote the social norm of going to bed on time<br>• Explore possibilities in national trajectories
Non-governmental organizations	• Create awareness among general practitioners (GPs) about the potential consequences of impaired parental mental health on children's sleep<br>• Create awareness among GPs in the general practitioners care practice about the importance of signaling and diagnosing unhealthy sleep, as this could help to identify other underlying health problems<br>• Create an overview of existing programs and services to simplify referrals<br>• Public libraries incorporate healthy sleep hygiene practices in their reading activities for children

References

1. Bonuck KA, Schwartz B, Schechter C. Sleep health literacy in head start families and staff: exploratory study of knowledge, motivation, and competencies to promote healthy sleep. Sleep Health. 2016;2(1):19-24.
2. Weinstein N. The Precaution Adoption Process. Health psychology: official journal of the Division of Health Psychology, American Psychological Association. 1988;7(4):355-86.
3. Hatton REM, Gardani M. Maternal perceptions of advice on sleep in young children: How, what, and when? British journal of health psychology. 2018;23(2):476-95.
4. Montaño DE, Kasprzyk D. Theory of reasoned action, theory of planned behavior, and the integrated behavioral model. Health behavior: Theory, research and practice. 2015;70(4):231.
5. Allen SL, Howlett MD, Coulombe JA, Corkum PV. ABCs of SLEEPING: A review of the evidence behind pediatric sleep practice recommendations. Sleep medicine reviews. 2016;29:1-14.
6. Ajzen I. From intentions to actions: A theory of planned behavior. Action control: Springer; 1985. p. 11-39.
7. Belmon LS, Brasser FB, Busch V, van Stralen MM, Harmsen IA, Chinapaw MJM. Perceived Determinants of Children's Inadequate Sleep Health. A Concept Mapping Study among Professionals. International journal of environmental research and public health. 2020;17(19).
8. Belmon LS, Busch V, van Stralen MM, Stijnman DPM, Hidding LM, Harmsen IA, et al. Child and Parent Perceived Determinants of Children's Inadequate Sleep Health. A Concept Mapping Study. International journal of environmental research and public health. 2020;17(5).
9. Belmon LS, van Stralen MM, Busch V, Harmsen IA, Chinapaw MJM. What are the determinants of children's sleep behavior? A systematic review of longitudinal studies. Sleep medicine reviews. 2019;43:60-70.
10. Walsh AD, Hesketh KD, Hnatiuk JA, Campbell KJ. Paternal self-efficacy for promoting children's obesity protective diets and associations with children's dietary intakes. The international journal of behavioral nutrition and physical activity. 2019;16(1):53.
11. Heerman WJ, Taylor JL, Wallston KA, Barkin SL. Parenting Self-Efficacy, Parent Depression, and Healthy Childhood Behaviors in a Low-Income Minority Population: A Cross-Sectional Analysis. Maternal and child health journal. 2017;21(5):1156-65.
12. Bandura A. Social foundations of thought and action: A Social Cognitive Theory. NJ: Prentice Hall: Englewood Cliffs; 1986.
13. Janz NK, Becker MH. The health belief model: A decade later. Health education quarterly. 1984;11(1):1-47.
14. Belmon LS, Komrij NL, Busch V, Oude Geerdink E, Heemskerk DM, de Bruin EJ, et al. Correlates of inadequate sleep health among primary school children. J Sleep Res. 2021:e13483.
